# Shape-programmed 3D printed swimming microtori for the transport of passive and active agents

**DOI:** 10.1038/s41467-019-12904-0

**Published:** 2019-10-30

**Authors:** Remmi Danae Baker, Thomas Montenegro-Johnson, Anton D. Sediako, Murray J. Thomson, Ayusman Sen, Eric Lauga, Igor. S. Aranson

**Affiliations:** 1 Department of Material Science and Engineering, The Pennsylvania State University, University Park, PA 16802 USA; 2 School of Mathematics, University of Birmingham, Birmingham, B15 2TT UK; 3 Department of Mechanical and Industrial Engineering, University of Toronto, Toronto, ON M5S 3G8 Canada; 4 Department of Chemistry, The Pennsylvania State University, University Park, PA 16802 USA; 5 Department of Applied Mathematics and Theoretical Physics, University of Cambridge, Cambridge, CB3 0WA UK; 6 Department of Mathematics, The Pennsylvania State University, University Park, PA 16802 USA; 7 Department of Biomedical Engineering, The Pennsylvania State University, University Park, PA 16802 USA

**Keywords:** Self-assembly, Metamaterials

## Abstract

Through billions of years of evolution, microorganisms mastered unique swimming behaviors to thrive in complex fluid environments. Limitations in nanofabrication have thus far hindered the ability to design and program synthetic swimmers with the same abilities. Here we encode multi-behavioral responses in microscopic self-propelled tori using nanoscale 3D printing. We show experimentally and theoretically that the tori continuously transition between two primary swimming modes in response to a magnetic field. The tori also manipulated and transported other artificial swimmers, bimetallic nanorods, as well as passive colloidal particles. In the first behavioral mode, the tori accumulated and transported nanorods; in the second mode, nanorods aligned along the toriʼs self-generated streamlines. Our results indicate that such shape-programmed microswimmers have a potential to manipulate biological active matter, e.g. bacteria or cells.

## Abstract (web summary)

While there are many demonstrations of self-propelled synthetic particles, there are fewer realisations of multimode swimming for the same particle. Here the authors demonstrate two swimming behaviours in magnetically manipulated microtori and show that these can manipulate other active particles.

## Introduction

Active systems consist of self-propelled agents, e.g. motile organisms or synthetic particles, that convert energy into mechanical movement. The intrinsic out-of-equilibrium nature of active systems leads to complex behaviors which often cannot be captured by well-established thermodynamic description. Examples of active matter include bacterial suspensions^1,2^, starling murmurations^3^, and fish schooling^4^.

Investigation of active matter systems in the limit of low Reynolds number, i.e. microscopic systems, has shown growing interest^5^. Microorganisms have been demonstrated to readily adapt to environmental changes. In particular, bacterial systems show a wide variety of complex behaviors, including spontaneous alignment in the presence of chemical gradients^6^ and altering rheological properties of the fluid^7–9^. Translating these complex behaviors to artificial systems is especially attractive for applications in fluid transport, small-scale mixing, and targeted cargo delivery^10,11^ but is hard due to intrinsic nanofabrications limitations.

Recent advances in self-propelled swimming particles have shown micro- and nanomotors are capable of biomimicking bacterial systems. For instance, chemically-powered bimetallic nanorods randomly swim in a manner somewhat akin to the swimming of microorganisms like *E. coli.*^12–16^. In addition, the micromotors (artificial swimmers) autonomously reorient to swim against flows, replicating biological rheotaxis behavior^17–22^. However, these “simple” artificial swimmers lack the swimming fidelity and multi-responsive behaviors of their biological counterparts, leaving much to be realized before using the micromotors for applications^12,23–31^.

Here we synthesize microscopic self-propelled tori using nanoscale 3D printing. We show experimentally and theoretically that the tori swimming behavior can be controlled by an applied magnetic field. We also demonstrate that the tori can manipulate and transport other artificial swimmers, bimetallic nanorods, as well as passive colloidal particles. To rationalize experimental observations, we developed a hydrodynamic and propulsion mechanism independent model to account for the new emergent phenomena in swimming microtori near boundaries. We determined two main mechanisms responsible for the swimming behavior: self-induced slip velocities across the surface and electrostatic potentials. Thus, our experimental and modeling insights can be applied to synthetic microswimmers powered by alternative mechanisms. In particular, our findings can be applied to biological systems by utilizing bio-compatible propulsion mechanisms, such as mounted enzymes^32^ or light^33^. These biocompatible, 3D printed microswimmers would then be able to interface and manipulate biological active matter, e.g. motile cells or bacteria–leading to the development of intelligent cell transport and therapy.

## Results

### Dynamic behaviors of microscopic tori

We 3D printed via two-photon lithography chemically-powered Janus (anisotropically patterned) platinum microtori with multi-responsive behaviors. The design was inspired by the “autophoretic torus”^34^; in turn by the “smoking ring” propulsion proposed by Purcell^35^ and later theoretically demonstrated in ref. ^36^. We found that microtori spontaneously broke symmetry to swim directionally across a surface at a constant velocity–radically different from the random swimming and tumbling behaviors seen in other micromotors, such as bimetallic nanorods or Janus spheres. See also Supplementary Note 1 and Supplementary Fig. 1 for more details on tori characterization.

We also demonstrated precise control for active cargo collection-transport and active swimmer manipulation by utilizing shape-encoded responses and surface functionalization to program specific behaviors in the tori. Specifically, we deposited a nickel binding layer to tune the tori’s axial orientation and as a result alter the particles’ behavior. This simple external control of the orientation (and by extension the behavior) of the tori gives rise to two distinct mobility modes: the first mode comprises horizontally oriented and linearly translating tori. The second mode represents the vertically oriented tori that translate linearly or cycloidally. We experimentally demonstrated the ability to manipulate other active particles, bimetallic and self-propelled nanorods. In the first mode the horizontally oriented tori actively collected and transported bimetallic nanorods to specified locations. In the second mode, the vertically oriented tori manipulated the swimming behavior of the bimetallic nanorods by aligning nearby nanorods along the tori’s streamlines.

### Chemically powered microscopic tori

**Fig. 1:**
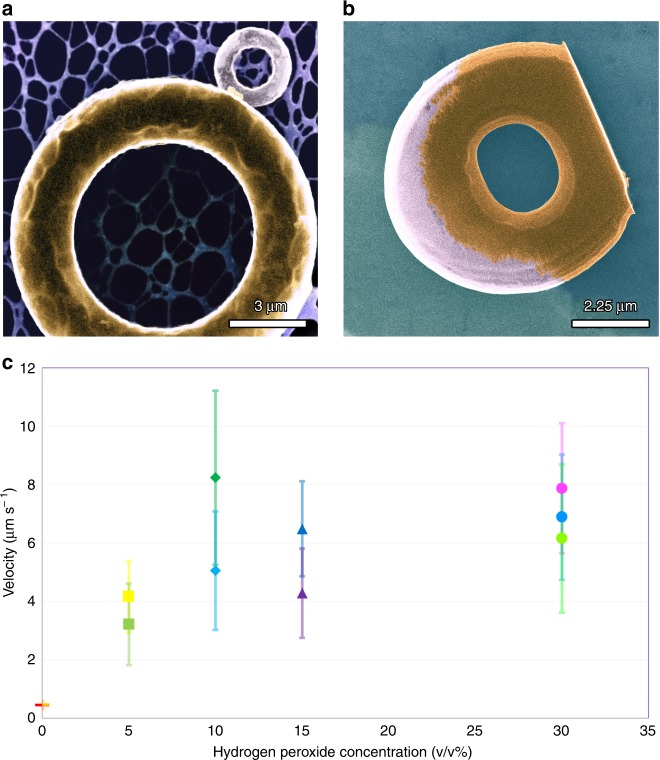
Tori with two types of surface coating. **a** A High-resolution transmission electron microscope (STEM) image of “glazed” Janus torus on a carbon lacey TEM grid. The metallic cap has been applied to the top of the tori. The scale bar is 3 μm. **b** SEM image of a “dipped” Janus tori on a carbon TEM grid. The bottom of the torus is “cut off” at the bottom to provide a stable base during printing and the metal evporation. The scale bar is 2.25 μm. **c** A graph representing the propulsion velocity dependence on the concentration of hydrogen peroxide. Red and orange horizontal bars are respectively the propulsion velocities glazed and dipped tori (with 40 nm Ni and 10 nm Pt) on a gold substrate at 0% H_2_0_2_. Green and yellow squares are respectively the propulsion velocities glazed and dipped tori (with 40 nm Ni and 10 nm Pt) on a gold substrate at 5% H_2_0_2_. Dark green diamond is the propusion velocity of glazed tori (with 40 nm Ni and 10 nm Pt)on a glass substrate at at 10% H_2_0_2_; and comparitively,teal diamond is the propulsion velocity of dipped tori (with 40 nm Ni and 10 nm Pt) on a gold substrate at 10% H_2_0_2_. Purple and dark blue triangles are, respectively, the propulsion velocities glazed (with 40 nm Ni and 10 nm Pt) and dipped tori (with 10 nm Ni and 40 nm Pt) on a gold substrate at 15% H_2_0_2_. Magenta, lime, and ice blue circles are the propulsion velocity of 3 μm diamater glazed tori, 7 μm glazed tori, and 7 μm dipped tori at 30% H_2_0_2_. The three tori have a coating of 40 nm Ni and 10 nm Pt. A full legend can be found in Supplementary Fig. 3 and in Supplementary Note 2. The error bars represent a unique standard deviation for that particular sample

The microtori were 3D printed via two-photon lithography on a Nanoscribe Photonic Professional GT system (see methods for fabrication details). After developing, we functionalized one face of the tori using electron beam metal evaporation; first depositing a thin binding nickel layer and then a catalytically active thin film of platinum. We investigated two different surface functionalizations: top “glazed” and sideways “dipped” Janus tori (Fig. 1a). See Supplementary Note 1 for additional physical characterization of “glazed” and “dipped” Janus tori.

**Fig. 2:**
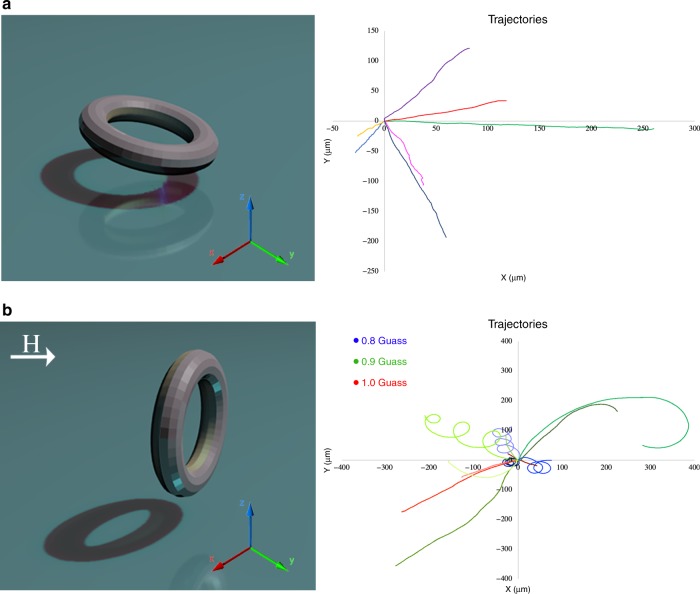
Swimming trajectories. **a** Extracted and re-centered trajectories for linearly translating dipped and glazed Janus tori. **b** Trajectories of vertically oriented tori in an increasing magnetic field. The trajectories change continuously from cyclodial to linear. Blue trajectories are for 0.8 G; green trajectories are for 0.9 G; and red trajectories are for 1.0 G

We extracted the printed particles via pipetting and then dropcast the colloidal solution onto a gold-coated glass slide. In water, the tori exhibit pure Brownian diffusion. However, in the presence of hydrogen peroxide, the 7 μm glazed and dipped tori consumed the fuel and directionally translated across the surface (Fig. 2a) (See Supplementary videos 1, 2). The slight decrease in velocity from glazed to dipped is the result of a decrease in the catalytic surface area on the torus. The propulsion velocity of the tori scales linearly with the concentration (v/v%) of hydrogen peroxide up to 10% and then saturates from 10 to 30% (Fig. 1b) . In 30% hydrogen peroxide, the glazed and dipped tori swam with the speed $\nu$ $\sim$ 7 μm s${}^{-1}$ (Fig. 1b).

Additionally, the deposition of a magnetic nickel binding layer (between the polymer and platinum) allowed us to alter the orientation of the swimming tori with a magnetic field varying between 0.8 and 1.0 G. In the presence of a magnetic field, the tori reoriented their axes to be perpendicular ($\phi \sim$ 90°) to the substrate. For a weaker magnetic field $\sim$0.8 G, their trajectories are cycloidal (Fig. 2b) (Supplementary Video 3). As the strength of the field increased to $\sim$1.0 G, the vertically-oriented tori rapidly altered their trajectories to be linear (Fig. 2b). We observed swimming trajectories in all directions, ruling out the migration in the magnetic field gradient.

Furthermore, the vertically oriented ($\phi$ $\sim$ 90°) glazed tori swam significantly faster ($\nu$ $\sim$ 16.36 $\pm$ 2.49 μm s${}^{-1}$) when compared to their horizontal orientation, see Fig. 1d. The increased velocity may be understood by considering both the increased drag present near a surface, and the direction of the propulsive force exerted by the torus; for perpendicular tori, all the propulsive force is used for  linear translation, whereas for the gliding (horizontally-oriented) tori, a significant fraction of the force is used to hover over the boundary while only a small part on the order $\sin (1{5}^{\circ })=0.26\approx 1/4$ is used for linear translation. See also Supplementary Note 1 and Supplementary Fig. 2 for more details on swimming behavior characterization.

**Fig. 3:**
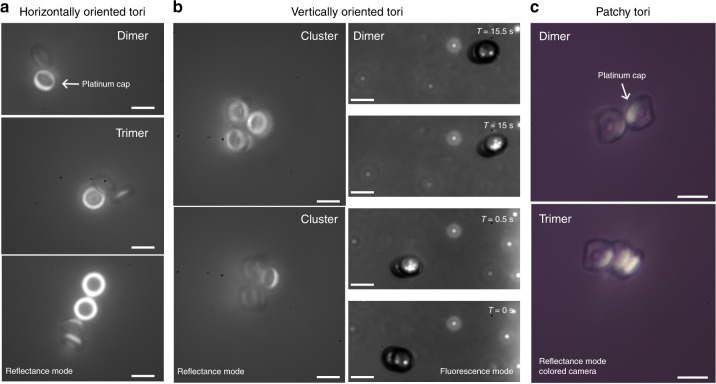
Collective states **a**–**b** Images of horizontally oriented and vertically oriented Janus tori clusters. Horizontally-oriented Janus particles form dynamic, unstable clusters. The vertically-oriented dimers swim in three dimensions. **c** An image of clustering, active patchy (dipped) tori. The polymer is transparent, and the metallic patches are reflected brightly in white. The dipped tori attach to nearby particles at the catalytic cap. All scale bars are 6.5 μm

We also observed formation of dynamic collective states of many active tori when in close proximity. Similar to bimetallic nanorods and other Janus particles^37^, Janus (glazed) tori formed stable dimers, and the inclusion of more Janus tori led to the formation of dynamic and unstable clusters (Fig. 3a, c). Trimers, tetramers, and similarly larger clusters spontaneously changed configurations, ejected individual particles, and demonstrated quasi 2D rotational and translational motion (Supplementary Videos 4, 5). For a complete study, we also investigated the behavior of vertically oriented Janus tori. We found that Janus tori on overlapping trajectories can collide, slide across each other’s surfaces, and form stable tumbling dimers, Fig. 3b. Interestingly, stabilized vertical-dimers showed a combined rotational and translation motion in 3D (Supplementary Video 6). Similar to their horizontally oriented counterparts, large clusters of the vertical Janus tori show dynamic states with different conformations.

We also analyzed swimming patchy (dipped) tori. Tori with a thick nickel layer (40  nm) formed stable dimers, trimers, and larger clusters. The dipped tori attach to other particles at the catalytic cap, a result arising from the strong magnetic dipoles, Fig. 3c. By contrast, in glazed tori with a thin nickel layer (10 nm), we observed active particles largely avoiding agglomerations. Our observation of the lack of clustering is similar to ref. ^38^, who used a softly magnetic binding layer to induce contactless particle-particle interactions due to repulsion of parallel magnetic dipoles.

### Propulsion and fluid transport mechanisms

We identified the dominant self-propulsion mechanisms that converted the chemical energy in the surrounding hydrogen peroxide into linear and cyclodial swimming motions. First, our microscopic tori retained a slight negative charge arising from the polymer and are repelled to an equilibrium distance away from the negatively-charged substrate. This electrostatic repulsion prevents the particles from irreversibly adhering to the substrate.

Furthermore, we assumed from previous Janus and patchy particle studies that the microscopic tori were self-electrophoretic^25,39^. We tested the hypothesis for a dominant self-electrophoretic propulsion mechansism by first including ~ 10 nM of salt (sodium chloride) into the hydrogen peroxide solution. The addition of salt inhibited self-electrophoretic swimming, and thus particles would then only move by diffusiophoresis. When we performed this experiment, we observed Brownian-like diffusive motion of the previously swimming microscopic tori and thus confirmed our hypothesis that the dominant propulsion mechanism is self-electrophoresis.

In addition to the self-electrophoretic propulsion mechanism, we compared diffusion and  advection driven fluid transport around the swimming tori. We used the dimensionless solute Péclet number, ${\rm{Pe}}=Lu/D$, to relate the importance of advection to diffusion. Here $L$ is the characteristic length, $u$ is the fluid flow velocity, and $D$ is mass diffusion coefficient of the solute. Here, the characteristic length $L\approx 3{{-}}7$  μm is the diameter of the tori, the flow velocity $u\approx 10{-}20$  μm s${}^{-1}$ is the propulsion velocity of the tori, and $D\approx 1{0}^{3}$  μm${}^{2}$ s${}^{-1}$ is the diffusivity of hydrogen peroxide in water. The estimate provides ${\rm{Pe}}\approx 0.1{-}0.2$. The results indicated that diffusion dominates the fluid advection around the tori^40^.

### The onset of linear motion

Most synthetic swimmers display random walks unless their rotations are quenched^19,41^ or their pathways are directed with external forces. However, we observed linear trajectories in swimming microscopic tori. Activating the microtori with hydrogen peroxide initiated fluid flows through the inner radius, i.e. donut hole, and outer radius that resulted in the particles hovering above the surface at a height $h$ $\sim$ 0.5-1  μm. We hypothesized that the hovering "parallel” orientation is intrinsically unstable. Any small fluctuations in the environment, e.g. thermal noise, led to a small perturbation of the hovering parallel orientation of the tori. The small perturbation in the orientation, combined charge redistribution across the tori, and generation of electro-osmotic flow created an instability surrounding the tori. The instability led to a spontaneous symmetry breaking^42,43^ across the tori during which the tori tilted to a stable vertical angle. The exact vertical angle is determined by the electrostatic potentials between the charge substrate and the anode-cathode across the catalytically active tori. The stabilized vertical angle, combined with a lack of rotational diffusion, gave rise to linear and persistent trajectories.

### Origin of the cyclodial trajectories

We attributed cycloidal trajectories of vertically swimming tori to a misplacement of the “center of propulsion” and the “center of drag” due to fabrication imperfections. In the presence of a permanent magnetic field $H$, the equations for the torus’ center of mass (COM) can be written in the dimensionless form:

1 $$\dot{x}={V}_{0}\cos (\phi )$$

2 $$\dot{y}={V}_{0}\sin (\phi )$$

3 $$\dot{\phi }=\omega +H\sin (\phi )$$

here $x,y$ represent the coordinates from the COM; $\phi$ is the in-plane angle; ${V}_{0}$ is the self-propulsion speed; and $\omega$ is the dimensionless rotation frequency due to torque that arose from the misalignment of the drag and propulsion forces, $H\sin (\phi )$ represents the magnetic torque exerted from the field onto the torus. We assumed for definiteness that the torus magnetic moment was oriented parallel to the torus axis of symmetry. Eq. (3) described two regimes of motion. In the first regime where $H\, > \, \omega$, the angle $\phi$ approached a stationary angle ${\phi }_{{\rm{s}}}$ that is defined in terms of $H,\omega$: $\sin ({\phi }_{{\rm{s}}})=\omega /H$. As a result, the COM moved in straight lines, as observed in experiments. In the second regime where $H\, <\, \omega$, the trajectories became cycloids because of the juxtapoised translational and rotational motion. Then the averaged COM drift velocity ${V}_{y}=\langle \dot{y}\rangle$ was obtained analytically for $0\, <\, H<\omega$ using Eq. (3): ${V}_{y}=\langle \dot{y}\rangle =\frac{1}{T}{\int }_{0}^{T}\dot{y}{\mathrm{d}}t$.

We used the period $T$ from Eq. (3) and also assumed from symmetry $\langle \dot{x}\rangle =0$. The we solved analytically last Eq. (3) and found the period $T=2\pi /\sqrt{{\omega }^{2}-{H}^{2}}$. The period $T$ diverged for $H\to \omega$ and approached $2\pi /\omega$ for $H\to 0$. For average velocity ${V}_{y}$ one obtained:

4 $${V}_{y}=\langle \dot{y}\rangle =\frac{1}{T}{\int }_{0}^{T}\dot{y}{\mathrm{d}}t=\frac{1}{T}{\int }_{0}^{2\pi }\frac{{\mathrm{d}}y}{{\mathrm{d}}\phi }{\mathrm{d}}\phi ={V}_{0}\frac{\sqrt{{\omega }^{2}-{H}^{2}}-\omega }{H}$$

Thus, from Eq. (4), we found the averaged drift velocity was ${V}_{y}\to 0$ for $H\to 0$ and ${V}_{y}\to -{V}_{0}$ for $H\to \omega$. This result indicated that the averaged drift velocity was zero when there was no magnetic field present (due to circular motion), and when a magnetic field was applied, the drift velocity gradually approached the self-propulsion velocity with an increase of the magnetic field. For the values of magnetic fields $H\, > \, \omega$, a cycloidal motion was transformed into a straight line. Furthermore, the torus drifted perpendicular to the direction of the magnetic field. In the general case, when the magnetic moment and torus axis of symmetry are not parallel, the expression for the mean drift velocity Eq. (4) does not change; however the drift direction depended on relative magnetic moment orientation.

### Programmable swimming orientation

**Fig. 4:**
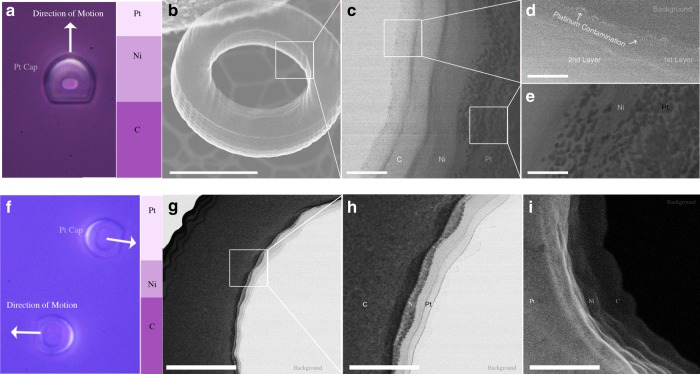
High-resolution scanning transmission electron microscopy (STEM) micrographs. **a** An image of the swimming orientation for tori with 40 nm nickel and 10 nm platinum. The Janus glazed  tori rest with their polymer structure near the substrate, and the patchy tori swim platinum-leading face forward. **b** A secondary-electron STEM image focused on the cross section of a single layer in the 3D printed structure. The scale bar is 3.25 μm. **c** A secondary-electron STEM image of the different materials deposited on separate layers of the tori. The nickel forms a smooth structure while the platinum nucleates into droplets on the binding layer. The scale bar is 300 nm. **d** A bright-field image of platinum contamination (in white) on the first polymer layer in the tori. The platinum contamination is responsible for an enhanced conductivity on the surface of the polymer. The scale bar is 200 nm. **e** A dark-field image of the platinum droplets on the uniform nickel binding layer. The platinum formed droplets $\sim$20 nm in diameter. The scale bar is 100 nm. **f** An image of the swimming orientation for tori with 10 nm nickel and 40 nm platinum. The Janus tori rest with their metal cap near the substrate, and the patchy tori swim polymer-leading. **g** Cross-sectioned, dark-field image of a tori coated in 10 in nickel and 40 nm platinum. We see a clear separation between the carbon polymer, nickel binding layer, and catalytic platinum layer. The scale bar is 700 nm. **h** A close-up, dark-field image at the interfaces between platinum and and nickel. We observe a smooth platinum layer ~200 nm thick. **i** Secondary-electron imaging (on STEM) of the smooth platinum layer. The platinum layer covered almost entirely the nickel binding layer. The scale bar is 200 nm

Traditionally, most self-electrophoretic Janus micromotors, namely microspheres, swim with the polymer-leading^19,41^. Herein, we have observed two preferred swimming orientations, i.e. platinum or polymer face leading. We have found through high-resolution transmission electron microscopy defects in the atomic interfaces among the polymer, nickel, and platinum that mainly contributed to the preferred swimming orientation (Fig. 4a, f). Our findings indicate that we can program preferred swimming orientation by purposefully including atomic scale defects in the thin films.

For tori that had $\sim$10 nm platinum layer deposited, we found a distinct lack of an anticipated uniform thin film. Instead, we observed $\sim$20 nm diameter platinum droplet features spattered across the nickel thin film (Fig. 4c–e). Gaps between the platinum clustering allowed the exposed nickel surface to rapidly oxidize when exposed to atmosphere or hydrogen peroxide (Fig. 4e). We concluded that the presence of surface oxidized nickel altered the catalytic properties and electron mobility during self-electrophoresis^44–47^ through 180${}^{\circ }$ flipping the positions of the anode-cathode in the tori.

In contrast, for tori where a $\sim$40 nm platinum layer was deposited, we observed the formation of a thin platinum film with three distinct grain boundaries (Fig. 4g–i). The platinum thin film completely covered the binding nickel layer; leaving nothing to be oxidized at atmospheres or in solution. Hence, tori with a uniform platinum film covering the nickel swam preferentially polymer side forward, as observed experimentally and previously reported in other Janus swimmers^19,41^.

### Controlled collodial crystal formations

In close proximity and in moderately dense solutions, Janus particles can form colloidal crystals^48–50^. We demonstrated precise control over the microtori to form disordered aggregates or colloidal crystalline structures by utilizing the magnetic field to control their individual swimming behaviors. In the absence of a magnetic field, the microscopic tori translate across the substrate and may collide with neighbors to form stable dimers. The dimers can evolve into trimers, tetrameters, and larger structures (3A). Similar to previous Janus colloidal crystals studies, we also observed dynamic interactions arising in trimers and larger structures due to the coupled electro- and hydrodynamics and self-generated chemical gradients.

With a magnetic field, we enforced the tori’s orientation to be perpendicular to the substrate and consequently also altered the swimming behaviors from linear to cyclodial. The change in microtori orientation and behavior led to the formation of collodial crystalline structures with rich dynamic behaviors. In particular, vertically-oriented microtori form dimers with three-dimensional helical swimming patterns (Fig. 3b). We formed ordered, crystalline structures with the addition of more microtori.

### Passive cargo manipulation

**Fig. 5:**
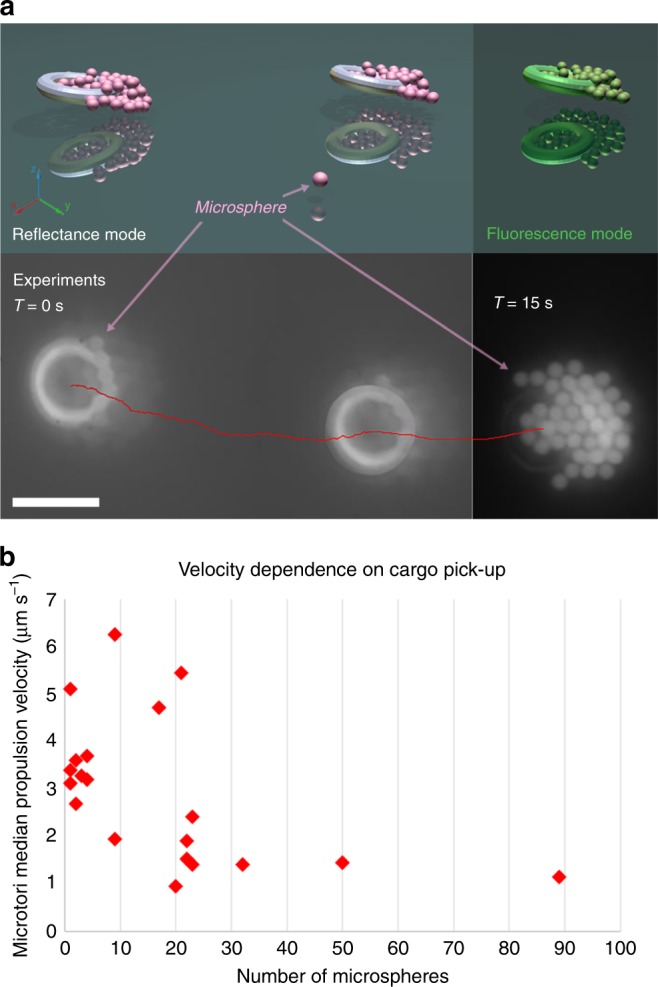
Passive cargo transport by swimming tori. **a** A time lapse sequence of a Janus donuts accumulating 1 μm sulfated polystyrene microspheres along the anode. The microspheres formed a hexagonal close-packed cluster around the inner and outer radii. The scale bar is 6.5 μm. **b** The propulsion velocity of the swimming tori versus number of attached microspheres

We also investigated the abilities of the swimming microscopic tori to pick-up and deliver cargo to specific sites. We used positively and negatively-charged microspheres as passive cargo. More specifically, we chose charged cargo to test the abilities of the tori to sort cargo based on charge. We observed the tori sorting cargo based on charge (Fig. 5a) (See Supplementary videos 7, 8). The tori preferentially collect negatively charged 1 μm polystyrene microspheres along the leading anode. Conversely, the tori accumulate  2 μm positively charged latex spheres along the cathode. Notably, the tori reached a critical accumulation of microspheres and then arranged the microspheres into a “halo” around the outer circumference. At this critical cargo concentration, the propulsion velocity steeply dropped. However, for tori that continued to swim past this critical cargo concentration, we found that their velocities were then largely independent of the cargo concentration, Fig. 5b. Finally, we utilized a strong external magnetic field to precisely direct tori carrying cargo. We also applied a magnetic pulse to release the cargo on-demand (See Supplementary video 9).

### Active cargo manipulation

**Fig. 6:**
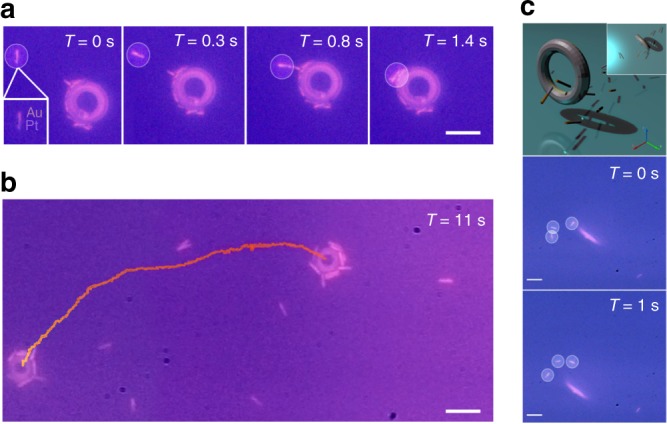
Active cargo transport by swimming tori. **a** A timelapse showing the hydro- and electrodynamic attachment of a swimming bimetallic nanorod to a microtori. The scale bar is 7 μm. **b** A timelapse showing a 3 μm diameter tori transporting numerous bimetallic nanorods. The scale bar is 2.5 μm. **c** A cartoon showing the vertical orientation of the micoscopic tori relative to the bimetallic nanorods near the surface. The bimetallic nanorods align along the self-generated fluid streamlines of the microscopic tori when nearby. The scale bar is 2.5 μm

For possible applications in living cell transport and sorting, we explored the complex task of manipulating and transporting active matter, i.e. other self-propelled microagents. We fabricated 2 μm long self-propelled, gold-platinum nanorods for their *E. coli* biomimicry^14^. We observed two distinct modes of active cargo manipulation that depended upon the orientation of the tori. In the first mode, horizontally-oriented tori actively collected swimming nanorods around their outer circumference and transport the active agents, Fig. 6 a, b and Supplementary video 10. We switched to a second mode of manipulation by orienting the tori perpendicularly to the substrate. In this vertical orientation the tori manipulated the swimming behaviours of the bimetallic nanorods, Fig. 6c. More specifically, the active nanorods oriented along the self-generated streamlines of the tori; either being pushed away from the tori along the outer radial streamlines or made to “jump through a [toroid’s] hoop.” (See Supplementary video 11). In addition, the tori can be continuously tuned between both modes by a magnetic field for a combination of active matter sorting and transport.

### Physical description of swimming torus dynamics

We modeled a single torus with an active surface (catalytic layer) oriented upwards and away from a substrate or plane boundary. We also varied the density $\rho$ of the torus; starting with ${\rho }_{a}=0$ representing a fluid density-matched (neutrally buoyant) swimmer. Far from the substrate, the neutrally buoyant swimmer swam vertically upwards, with decreasing speed as the density was increased. At a critical density ${\rho }_{c}$, the torus hovered, and at higher densities the torus sedimented.

**Fig. 7:**
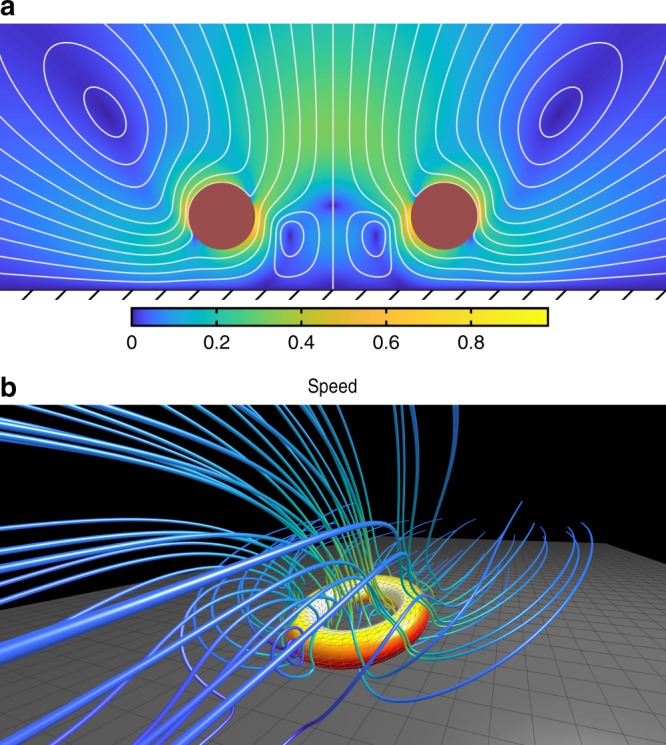
Flow streamlines generated by a patchy (glazed) torus. **a** Flow streamlines produced by an active, uncharged torus, hovering above a plane boundary, with nondimensionalized speed. **b** Flow streamlines and surface concentration of a steady gliding torus, at an angle of $1{2}^{\circ }$, shown in the frame in which the torus is stationary

**Fig. 8:**
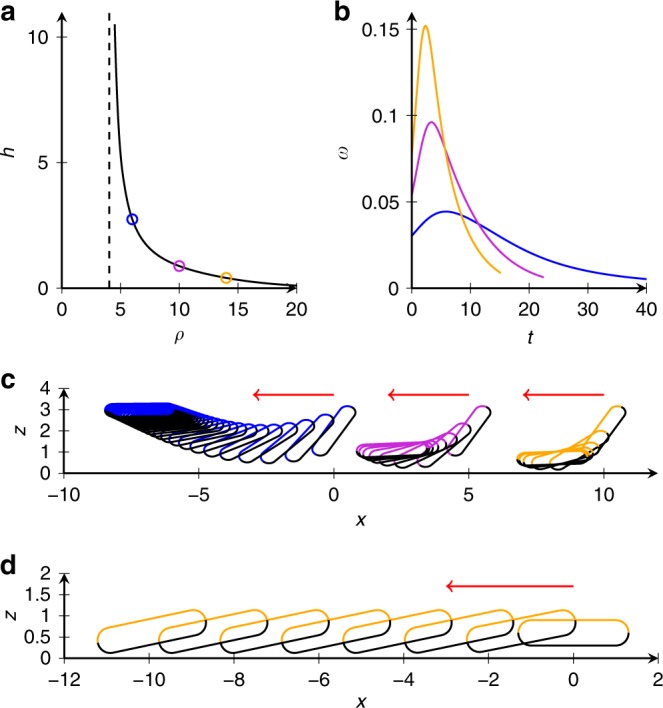
Simulated dynamics of tori over a plane boundary. **a** The height $h$ at which the base of a torus with unit activity and mobility hovers over the plane boundary as a function of its added density $\rho$ (where $\rho =0$ is neutrally buoyant). The asymptote $\rho =4.33$ is the density at which such a torus in free space neither sinks nor swims. **b** The relaxation to a stable horizontal hovering state of uncharged tori, showing decaying angular velocity $\omega$ with time. **c** Swimmer trajectories with equal intervals of uncharged tori. **d** Transition from horizontal to steady gliding state (at a 12${}^{\circ }$ angle) of a charged torus. Red arrows indicate progression of time. Here the major and minor radii of the tori ${a}_{1}=1,{a}_{2}=0.3$

The behavior of a sedimenting self-propelled torus changed significantly as it approached the plane boundary at $z=0$; the torus gained a propulsive boost by “pushing off” the substrate with the flow it generated. For densities above the critical density, the torus approached a critical height ${z}_{0}(\rho ),\rho \, > \, {\rho }_{c},$ where the swimmer began to hover above the substrate via self-propulsion (autophoretic slip flows) (Fig. 7a). We measured the heights above the plane boundary $h$ from the bottom of the torus as a function of density $\rho$. As the density of the torus increased we observed a decrease in the hovering heights (Fig. 8a).

We also considered the effect of electric charge on a hovering torus. In the absence of charge, a vertical position of a torus hovering via autophoretic slip flows above a plane boundary was stable. We perturbed the active torus to a non-zero angle and observed no appreciable changes in the slip velocity on the portion of the torus closest to the boundary. However, the portion of the torus closest to the boundary exerted more thrust than the other side and resulted in a restorative torque. We fully captured the relaxation to a stable configuration for different torus densities (Fig. 8b, c).

For the second case we considered a hovering torus near a boundary with charge. We included a dipolar charge distribution across the active surface; we also set a positive charge on the boundary due to surface charge absorption. The torque tilted the swimmer away from the parallel to perpendicular state (assuming there were no collisions with the plane boundary). Combined with the presence of a solute fuel-driving (diffusiophoretic) surface slip flow, the torus glided at a stable angle; the exact stable angle is determined from the competing electrostatic attraction/repulsion forces and restorative torque from the slip flow. We captured the dynamics of an initially horizontally-oriented torus that quickly reached a steady angle of 12${}^{\circ }$ as it glided across the surface (Fig. 8d). The flow streamlines and surface solute concentration are shown for the example in Fig. 7b. For simplicity, we did not use any short range repulsive potential; however, depending on the relative charge, short range repulsion may be what prevents the leading edge from adhering to the lower boundary.

## Discussion

We utilized two-photon lithography to 3D print and program by shape autonomous, multi-responsive behaviors in artificial microswimmers. We fabricated chemically-powered microscopic tori with nanoscale features; these tori had either half-coated (glazed or Janus) or patchy (dipped) platinum catalytic layers. In the presence of hydrogen peroxide, the tori instantaneously begin to hover above the surface because of the self-electrophoretic propulsion. Due to the charge instability, i.e. moving surface charges arising from self-electrophoresis, the tori spontaneously break symmetry and tilt to a stable angle. The tori then glide across the surface; eventually organizing into dynamic clusters that swim in three dimensions.

Furthermore, we programmed two modes, i.e. linear or cycloidial swimming behaviors, into the tori to perform complex tasks. For a simple task, the tori scavenge and sort passive and charged microspheres in solution; the tori organized the tracer particles into a hexagonal closed packing. At the same time, the swimming tori were also capable of sorting tracer particles across their anode-cathode. We demonstrated that the cargo can be delivered to a desired location and released on demand. From these results, it is conceivable to utilize our motile and programmable swimmers to clean freshwater of pollutants, such as microplastic particles.

We also tasked the microtori with a more complex objective–the manipulation of active matter. In the first mode the tori swam linearly and almost parallel to the substrate; the swimmers accumulated and transported very dense, self-propelled bimetallic nanorods. Switching to the second mode, the tori reoriented perpendicular to the substrate and manipulate the orientation of nearby nanorods. The bimetallic nanorods aligned along the self-generated fluid streamlines of the tori. The tori then either drew in through the center radii or pushed away nearby nanorods. To our knowledge, this is the first such manipulation of active matter by autonomous, artificial swimmers.

The concepts and results presented here can be further extended for biological applications. In particular there are many options available for biocompatible catalysts, including tethered enzymes or light-driving propulsion mechanisms. The addition of more complex external fields and chemical gradients can be used to indirectly and directly guide the autonomous swimmers. The tori could then be directed to deliver living cargo, such as cells, to specific sites for cell therapy; or collectively organize the tori to direct their flow for cellular transport and sorting.

## Methods

### 3D printing file generation

We generated the CAD files using Autodesk Fusion 360. We then chose a torus structure with a 100 mm diameter and 25 mm thickness. We exported the CAD files as a solid stl file. The stl file was then opened on the Describe–the Nanoscribe Photonic software for generating job and data files for 3D printing. We then selected the polymer (IP-Dip 63×) and scaled the tori down to a diameter of 3 or 7  μm. We then selected slices and hatches as 0.1 to generate the smoothest structure possible. We then generated an array of either 100 $\times$ 100 or 500 $\times$ 500 particles with a spacing of 8–20  μm between particles. The array was broken into smaller sections consisting of 10 $\times$ 10 particles. The particles in these smaller sections then printed as a single unit.

### Particle fabrication

The microparticles were 3D printed on a Nanoscribe Photonic Professional GT. Nanoscribe IP-DIP photoresist was dropped onto a cleaned DiLL glass slide and then loaded into the instrument at 63× oil immersion. Using two-photon lithography, we 3D printed microstructures from a computer-generated model. After exposure, the particles were developed in propylene glycol monomethyl ether acetate (PGMEA) or 1-methoxy-2-propyl acetate (SU-8 Developer) for 25 minutes and then exposed to isopropyl alcohol (IPA) for 5 min to stop the developing process. Then the microparticles were baked at 200 ${}^{\circ }$C for 10 min to solidify the structure.

We then evaporated the desired metals—nickel and platinum—onto the particles using a direct line-of-sight approach on a Semicore E-Gun Thermal Evaporator. The particles were mounted vertically into the instrument with kaplan tape and pumped down till vacuum pressure reach $\sim$1 $\times$ 10${}^{-6}$ or 1 $\times$ 10${}^{-7}$ Torr. We then evaporated the nickel binding layer at a rate 0.5–1.0 $\mathring{\rm{A}}$ s${}^{-1}$ at a pressure $\sim$1 $\times$ 10${}^{-6}$ Torr. Afterwards, we then evaporated platinum at 0.1–0.3 $\mathring{\rm{A}}$ s${}^{-1}$ at $\sim$1 $\times$ 10${}^{-6}$ Torr. We then allowed the system to cool for 30 min to an hour before removing the samples from the tool. Then the particles were extracted from the surface with pipetting. The colloidal dispersion of particles was collected for experiments. No further washing was required for the particles prior to experimenting.

### Experimental set up

A colloidal dispersion was dispensed onto a gold-coated slide. The gold coating prevented microparticles from adhering irreversibly to the glass surface. Then 5–30% hydrogen peroxide was introduced to the system to activate the catalytic particles. We protected the system from evaporation by sealing the droplet with a cover slip. Then the active particles were observed on an inverted Olympus Microscope IX83 at ×10–100 magnification—oil immersion was specifically used for ×60 and ×100 magnification—and recorded at 11–56 frames per second with an Allied Vision ProSilica GT GIGE camera.

### Electron microscopy

All scanning electron microscopy (SEM) was performed on a FEI Quanta 250 FEG. The samples were imaged directly on fabrication glass slides, mounted with double sided carbon tape, and the sample area grounded to the stage with copper foil tape to minimize charging during imaging.

For transmission electron microscopy analysis (TEM), samples were collected from the glass slides by pipetting, then drop coated onto holey carbon grids (Ted Pella 01881). The holey grids were selected as they allowed high-resolution imaging of the tori edges over vacuum in the TEM. All transmission and scanning transmission electron microscopy was performed on a Hitachi HF3300 ETEM set to 300 kV, 5 μA emissions current, STEM mode. The instrument was equipped with conventional TEM imaging, as well as secondary-electron scanning transmission electron microscopy(SE-STEM), bright field, (BF-STEM) and dark-field (DF-STEM) sensors. This setup generates three images: a topographical surface profile (SE- STEM), a diffracted image of the cross section (DF) with high elemental contrast, and a direct image of the cross section (BF). The BF and DF images are sensitive to the elements present, and clearly show different elements with different contrast. All files were captured in the DM3 Gatan format and post processed using ImageJ. The images were then recolored using Adobe Photoshop CC 2018.

### Numerical methods

A simplified mathematical model of the system whereby the torus dynamics are driven by surface slip flows proportional to the gradient of a self-generated concentration field was considered. Motivated by the result that swimming behavior is largely independent of particle size for the particles studied, we consider a zero Péclet number limit. This limit entails first solving Laplace’s equation for the concentration field, then solving the inertialess Stokes flow equations (suitable for microscale flows) with the gradient of the concentration field providing the slip velocity boundary condition for the flow.

We solved both Laplace’s equation and the Stokes flow equations using a regularized boundary element method^51^. The advantage of this method is that only the boundary of the torus need be meshed, and variables are only solved for on the domain boundary, rather than in the bulk. Thus 3D dynamic phoretic simulations are quick and require no remeshing^52^. For Laplace’s equation, we solved in the upper-half space $z \, > \, 0$, and we apply the no-flux boundary condition on $z=0$ and on the polymer portion of the swimmer. On the metal portion of the swimmer, we apply a simplified condition of constant flux. For the Stokes equations, we applied the no-slip condition ${\bf{u}}={\bf{0}}$ on $z=0$ and that slip velocity is proportional to the surface gradient of the concentration field over the whole surface. The no-flux, no-slip wall is incorporated into the boundary element framework via the method of images, removing the need for a computational mesh of the boundary and increasing numerical efficiency.

The torus was subject to a weight force acting in the negative $z$-direction, and may be subjected to a short-range repulsive potential acting in the positive $z$-direction that prohibits the swimmer passing through the wall $z=0$. Finally, the metal portion of the torus may be given a charge that varies across the surface, and was subject to electrostatic interaction with the wall $z=0$. Whilst this model has a number of simplifying assumptions, it is nonetheless able to effectively capture the physical processes in our system.

## Supplementary information

Supplementary Information

Description of Additional Supplementary Files

Supplementary Movie 1

Supplementary Movie 2

Supplementary Movie 3

Supplementary Movie 4

Supplementary Movie 5

Supplementary Movie 6

Supplementary Movie 7

Supplementary Movie 8

Supplementary Movie 9

Supplementary Movie 10

Supplementary Movie 11

## Data Availability

The experimental data, including additional videos not available through the current Supplementary information, can be requested through I.S.A.

## Code availability

Access to the simulation codes can be made available through TDM-J. Request for supporting 3D printing files should be submitted to R.D.B.
