# Supplementary material for: Shape-programmed 3D printed swimming microtori for the transport of passive and active agents
Source: Nat Commun. 2019 Oct 30;10:4932. doi: 10.1038/s41467-019-12904-0 (PMC6821728; doi:10.1038/s41467-019-12904-0)
Supplement: Supplementary file 1 — Supplementary Information [file 41467_2019_12904_MOESM1_ESM.pdf]

# Supplementary Information

Remmi Danae Baker et al.

## Supplementary Note 1: Tori Characterization.

**Physical Characterization** The nature of our electron metal evaporation only permitted direct line-of-sight metal deposition onto the fabricated donuts. By changing the orientation of the tori during fabrication, i.e. print horizontal or vertical, we could alter the catalytic patch of the particles. We fabricated two sets of active tori: half-coated platinum Janus or irregularly platinum patches (Supplementary Fig. 1A-B). Notably, the irregularly platinum tori are misshapen during the fabrication process, resulting in a flat edge. Furthermore, we analyzed the tori post-liftoff, i.e. removal from the substrate, and observed only minor damage to the overall structure (Supplementary Fig. 1 C).

**Swimming Behavior Characterization** We measured the swimming velocities for both horizontally- and vertically-oriented tori (Supplementary Fig. 2 A-B). Defective particles–fabrication flaws easily seen under the microscope–are excluded from the distribution. Afterwards, we truncated the velocity distributions to the first population point, i.e. we do not show empty space before the distribution. The velocity distributions were broadened because we do not screen for nearby particles colliding or localized fuel depletion from actively swimming neighbors; both factors contribute heavily to a decrease in the swimming velocities.

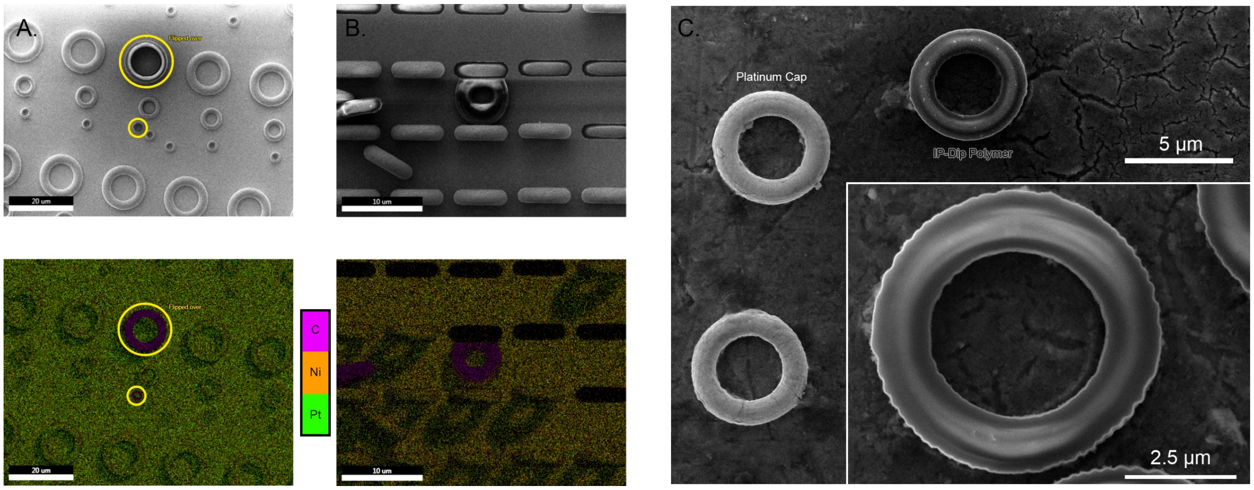


Supplementary Figure 1: **Microstructure of the tori revealed by electron microscopy.** (A) Top and bottom images are scanning electron microscopy (SEM) and energy dispersive spectroscopy (EDS) for a 3-D printed Janus tori coated with a binding nickle and catalytic platinum layer. Outlined in yellow is a tori that flipped over to exposed to the polymer side. (B) Similarly, platinum patchy tori imaged and elementally mapped. For both patchy and Janus tori, a uniform nickel and platinum coating is observed. (C) SEM images of fabricated Janus donuts on a steel stub. We observed a smooth polymer structure (in dark grey) coated with uniform platinum cap. There is minor rippling along the equator of the donut from the rastering of 3-D printed layers. The nickel binding layer is only exposed along the equator and is otherwise capped by the platinum catalyst.

We also measured the vertical angle for all horizontally-oriented tori over the entire trajectory (Supplementary Fig. 2 C). We conducted the measurement primarily by comparing the estimated radius to the actual radius (found on SEM). We then took the cosine of the estimated to actual radius and found $\phi \sim 15^\circ$ (Supplementary Fig. 2 D). As a secondary check, we measured the height (from top to bottom of the tori) by hand at 100x oil-immersion. We then took the sine of our height and estimated radius and found agreement for $\phi \sim 15^\circ$. We primarily see a narrow peak at $\phi \sim 15^\circ$. We truncated to exclude smaller peaks at $\phi \sim 10^\circ$, $16^\circ$. These smaller peaks are the result of defective donuts.

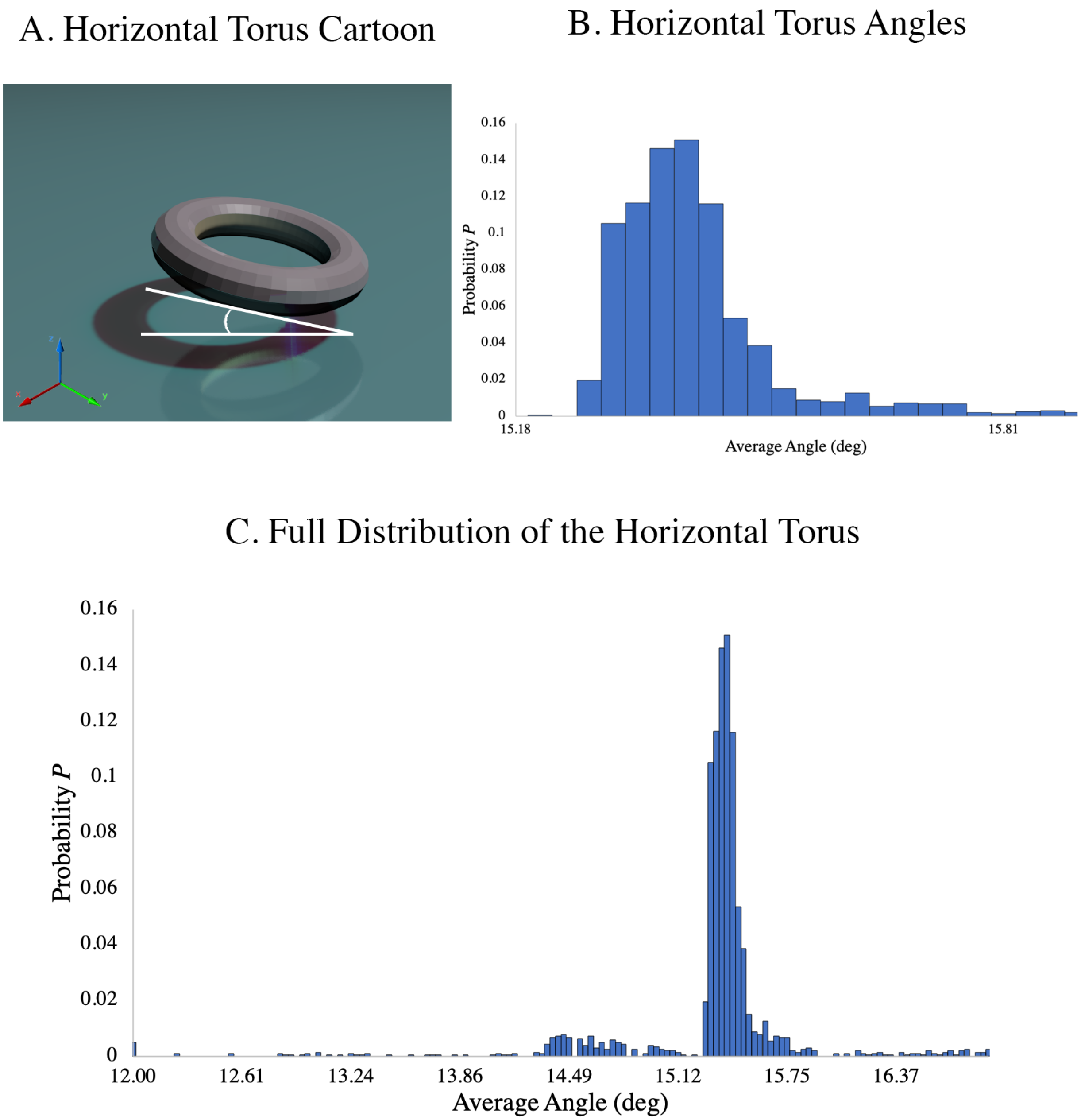


Supplementary Figure 2: **Vertical Angle Distributions.** (A) A cartoon illustrating the measurement of the vertical angle next to the substrate. (B) A truncated histogram of the vertical angles. (C) The full histogram of the vertical angles.

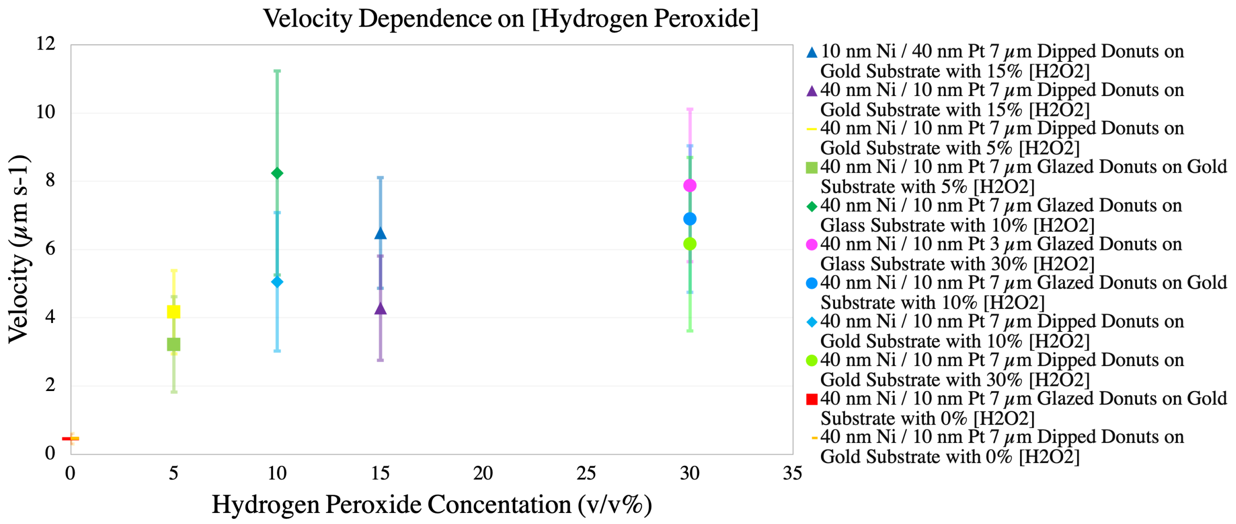


Supplementary Figure 3: **Velocity Dependence on Hydrogen Peroxide.** The graph relating the dependence of velocity to the concentration of hydrogen peroxide. We included the legend.

**Supplementary Note 2: Additional Experimental Graphs.**

In our study, we analyzed the velocity dependence on the concentration of hydrogen peroxide. Here we have included an additional velocity vs hydrogen peroxide concentration graph (Supplementary Fig. 3) with the legend attached. The graph is otherwise identical to the graph presented in Figure 1B of the main manuscript.
