# Supplementary material for: Shape-programmed 3D printed swimming microtori for the transport of passive and active agents
Source: Nat Commun. 2019 Oct 30;10:4932. doi: 10.1038/s41467-019-12904-0 (PMC6821728; doi:10.1038/s41467-019-12904-0)
Supplement: Supplementary file 2 — Description of Additional Supplementary Files [file 41467_2019_12904_MOESM2_ESM.pdf]

Description of Additional Supplementary Files

File Name: Supplementary Movie 1
Description: Horizontally-oriented "glazed" Janus tori linearly translating across the substrate.

File Name: Supplementary Movie 2
Description: Horizontally-oriented ”dipped” Janus tori linearly translating across the substrate.

File Name: Supplementary Movie 3
Description: Vertically-oriented Janus tori swimming across the substrate in cyclodial trajectories. The magnetic field strength is ~ 0.8 G.

File Name: Supplementary Movie 4
Description: Horizontally-oriented Janus tori forming dimers.

File Name: Supplementary Movie 5
Description: Two vertically-oriented Janus tori colliding and forming a dimer that swims with both translational and rotational motion.

File Name: Supplementary Movie 6
Description: A vertically-oriented, swimming dimer captured at 100x under reflectance mode. A glass substrate was used to minimize the reflectance from a gold substrate.

File Name: Supplementary Movie 7
Description: Horizontally-oriented "glazed" Janus tori accumulating 2 µm positively charged tracer particles.

File Name: Supplementary Movie 8
Description: Vertically-oriented "glazed" Janus tori accumulating 1 µm negatively charged tracer particles. The tori are steered with a magnetic field.

File Name: Supplementary Movie 9
Description: Horizontally-oriented "glazed" Janus tori accumulated 1 µm negatively charged tracer particles. We applied a magnetic pulse to release the microspheres on demand.

File Name: Supplementary Movie 10
Description: Horizontally-oriented "glazed" Janus tori accumulating 2 µm long, self-propelled gold-platinum nanorods.

File Name: Supplementary Movie 11
Description: Vertically-oriented "glazed" Janus tori making 2 µm long, self-propelled gold-platinum nanorods jump through their "donut hole."
